# Dabigatran in the Treatment of Warfarin-Induced Skin Necrosis: A New Hope

**DOI:** 10.1155/2016/3121469

**Published:** 2016-03-27

**Authors:** Christos Bakoyiannis, Georgios Karaolanis, Nikolaos Patelis, Anastasios Maskanakis, Georgios Tsaples, Christos Klonaris, Sotirios Georgopoulos, Theodoros Liakakos

**Affiliations:** 1st Department of Surgery, Vascular Surgery Unit, Laikon General Hospital, Medical School of Athens, Agiou Thoma 17, 11527 Athens, Greece

## Abstract

Warfarin-induced skin necrosis is an infrequent and well-recognized complication of warfarin treatment. The incidence was estimated between 0.01% and 0.1% whereas a paradoxal prothrombotic state that arises from warfarin therapy seems to be responsible for this life-threatening disease. To the best of our knowledge we present the first case of an old woman diagnosed with warfarin-induced skin necrosis, in whom novel oral anticoagulants and extensive surgical debridement were combined safely with excellent results.

## 1. Introduction

Warfarin-induced skin necrosis (WISN) is an infrequent and well-recognized complication of warfarin treatment. In the literature, WISN incidence was estimated between 0.01% and 0.1% [1, 2]. The accurate pathogenetic mechanism is not clear and the paradoxal prothrombotic state that arises from warfarin therapy seems to be associated with the relative decrease in vitamin K-dependent clotting factors (e.g., protein C) or the hereditary deficiencies of protein S, Factor V Leiden, and antithrombin III [3, 4]. This imbalance can cause microthrombi which interrupt blood flow to the skin and cause necrosis. We present a case of a 72-year-old woman with skin necrosis after initiation of warfarin therapy due to atrial fibrillation and who was safely treated with novel oral anticoagulants (NOACs).

## 2. Case Presentation 

A 72-year-old woman was admitted to our department due to acute ischemia of the right leg. She was diagnosed with persistent nonvalvular atrial fibrillation 7 days ago, and it was decided that she would benefit from warfarin for stroke prophylaxis. Warfarin was prescribed at 2 mg daily as a slow loading dose and on the fifth day the primary care physician increased the dose to 5 mg, due to the low value of the international normalized ratio [INR = 1.5]. Moreover, the patient had a history of hypertension and diabetes mellitus and there was no history of any trauma or local/systemic infection.

**Figure 1:**
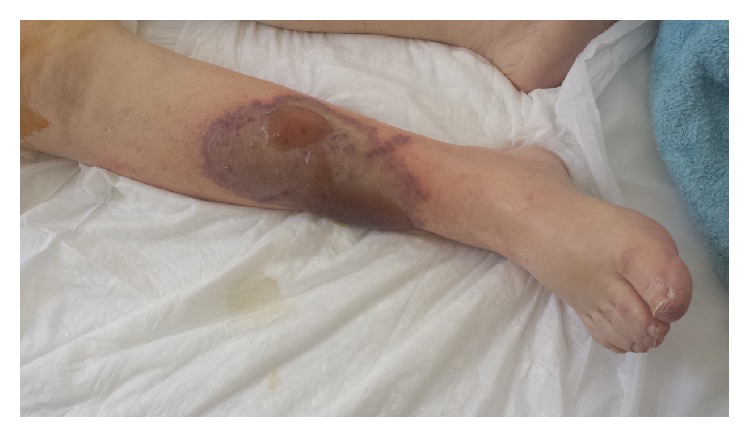
Patient's right leg at presentation to the vascular department.

On physical examination, the right leg of the patient was found to be pale and cold with the sign of developing mottling and cyanosis from knee level down. The calf muscles were also tender on examination. She had no detectable arterial pulses below her right knee by palpation, which was confirmed by an emergency Doppler examination revealing the complete lack of blood flow in both dorsalis pedis and posterior tibial arteries. Furthermore, a necrotic lesion was revealed measuring 5 cm in diameter on the lateral aspect of lower right leg [Figure 1].

**Figure 2:**
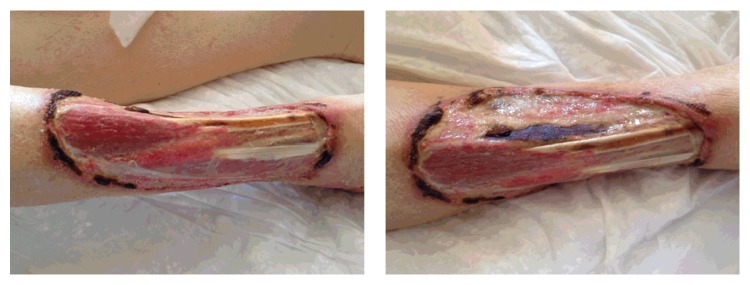
Surgical debridement.

The diagnosis of acute right leg ischemia due to the acute occlusion of the 3-infrapopliteal arteries was considered. A dose of intravenous (IV) heparin was chosen and lower limb thromboembolectomy (TE) was immediately performed via the right femoral artery. The patient's postoperative course was uncomplicated, and the extremity tenderness and mottled skin were improved. Moreover, dermatology consults and skin biopsy revealed noninflammatory thrombosis with focal necrosis in the leg lesion. Warfarin therapy was discontinued and the patient was started on dabigatran 150 mg twice daily. Skin lesion was followed with conventional surgical debridement and was seen to improve [Figure 2] in a few days, without any recurrence under dabigatran therapy.

## 3. Discussion

Warfarin skin necrosis has already been described in the literature [5, 6]. Breast, buttocks, abdomen, thighs, and calves are more susceptible probably because of the reduced blood supply to adipose tissue. The first case has been reported with breast necrosis [7] but Verhagen [8] was the first to describe the association between skin necrosis and oral anticoagulant treatment. Since then, middle-aged, overweight women have been reported in the literature, as cases of skin necrosis resulting from oral anticoagulant treatment [1, 3]. In some cases, skin necrosis had appeared within 10 to 15 days after the initiation of warfarin treatment [9] whereas more recent studies indicated that the most common time for appearance of the first symptoms of skin injury is considered to be 1 to 10 days after the initiation of the warfarin treatment [10]. Necrosis occurred in individuals above 50 years of age with International Normalized Ratio (INR) above 4. The precise pathological pathway remains unclear, and many hypotheses have been formulated.

Recently, several NOACs have been developed and offer potential advantages over vitamin K antagonists, such as rapid onset and offset action, absence of an effect of dietary vitamin K intake on their activity, and fewer drug interactions [11]. Randomized clinical trials [12–14] were performed comparing NOACs and warfarin as a treatment of choice in patients with atrial fibrillation and had promising and clear results. NOACs showed a favorable risk-benefit profile, with significant reduction in stroke, intracranial hemorrhage, and mortality. Treatment with anticoagulants in elderly patients requires weighing the serious risk of stroke against an equally high risk of major bleeding (gastrointestinal and extracranial bleeding). The use of NOAC should certainly not be withheld from elderly patients who have a clear indication for oral anticoagulation. However, caution is warranted for major bleeding due to the lack of specific antidote to rapidly reverse the anticoagulant's effect in this group of patients [15].

Based on these results, the authors chose two simultaneous ways to treat this patient. Surgical debridement and purification of the injured tissue were the first choice and administration of NOACs was the second. In the literature few reports on sporadic similar cases have been described [1, 3, 8]. However the skin extension was smaller and surgical debridement was not obligatory. Recently, 2 cases [16] with WISN have been published using only NOACs as a treatment option without surgical debridement. The authors came to the conclusion that NOACs can be safely used in this relatively rare but serious clinical situation. We report this case with WISN emphasizing on the fact that novel anticoagulants and surgical debridement could safely combine in this relatively rare but serious clinical situation with excellent results.

## 4. Conclusion

NOACs seem to be an effective solution in cases of WISN. We propose that no such complications might have been encountered if the first choice drugs were NOACs instead of the “old habit” of preferring warfarin.
